# Safety and Efficacy of Apixaban For Therapeutic Anticoagulation in Critically Ill ICU Patients with Severe COVID-19 Respiratory Disease

**DOI:** 10.1055/s-0040-1720962

**Published:** 2020-11-23

**Authors:** Eric Wenzler, Monaz H. Engineer, Maidah Yaqoob, Scott T. Benken

**Affiliations:** 1 Department of Pharmacy Practice, College of Pharmacy, University of Illinois at Chicago, Chicago, Illinois, United States; 2 Division of Pulmonary, Critical Care, Sleep, and Allergy, Department of Medicine, University of Illinois at Chicago, Chicago, Illinois, United States

**Keywords:** apixaban, coagulopathy, COVID-19, thrombosis, SARS-CoV-2

## Abstract

**Introduction** Despite the use of unfractionated heparin (UFH) or low molecular weight heparin (LMWH), rates of thromboembolic disease, and subsequent morbidity and mortality remain unacceptably high in patients with severe novel coronavirus disease 2019 (COVID-19) disease. Direct oral anticoagulants (DOACs), such as apixaban, have numerous purported benefits although the safety and efficacy of their use in intensive care unit (ICU) patients with severe COVID-19 has yet to be evaluated.

**Materials and Methods** Single-center, retrospective cohort study of 21 ICU patients with severe COVID-19 respiratory disease treated with apixaban for atrial fibrillation (AFib), venous thromboembolism (VTE), catheter-induced thrombosis, and/or COVID-19-induced coagulopathy. The primary objective was to evaluate the incidence of bleeding events and secondary objectives included thromboembolic events, coagulation parameters, and mortality.

**Results** Ninety percent of patients were non-White, 43% were obese, 90% had acute respiratory distress syndrome, and 76% required mechanical ventilation. Nearly half of (47.6%) also experienced renal dysfunction and required renal replacement therapy. Eighty-six percent of patients received prophylaxis or treatment with UFH or LMWH within the 24-hour period prior to apixaban initiation. Patients were initiated on apixaban for the treatment of suspected or confirmed VTE (67%) or AFib (33%). All coagulation parameters remained abnormal but stable throughout the 10-day monitoring period. No patients experienced any major bleeding events or thrombosis throughout the study period. There were four deaths during the follow-up period, all deemed unrelated to coagulopathy or bleeding.

**Conclusion** Apixaban appeared safe and efficacious in ICU patients with severe COVID-19 disease. These data encourage future trials seeking to optimize anticoagulation strategies in patients with severe COVID-19.

## Introduction

The novel severe acute respiratory syndrome-coronavirus-2 (SARS-CoV-2) and its associated disease novel coronavirus disease 2019 (COVID-19) have now decimated the U.S. healthcare system for more than 8 months causing over 240,000 deaths 1 while swiftly and unforgivably unveiling the devastating consequences of systemic viral infection on an unprecedented scale. Since the first reports emerging from Wuhan, China, the inexorable link between COVID-19 infection, hyperinflammation, and mortality were quickly established. 2

Infection with COVID-19 has been associated with an increased risk of venous and arterial thromboembolic disease due primarily to virus-induced hyperinflammation and subsequent widespread immunothrombosis. SARS-CoV-2 utilizes the angiotensin converting enzyme 2 (ACE2) receptor to infect its host, which is expressed throughout numerous bodily organs and by endothelial cells. Direct viral infection of the endothelium and diffuse endothelial inflammation result in thrombotic microangiopathies and the recruitment of host immune cells and proinflammatory cytokines, leading to pervasive endothelial dysfunction and apoptosis. 3 This dysfunction then promotes microvascular derangements and a shift in the vascular equilibrium toward a vasoconstrictive, inflammatory, and hypercoagulable state. Although this process is not unique to the SARS-CoV-2 virus, the systemic coagulopathies observed in patients with severe COVID-19 infection are noticeably distinct. Among these, an increased D-dimer concentration has been observed in almost 50% of patients with COVID-19-induced coagulopathy and has been consistently associated with an increased risk of death. 4 5 Conversely, only mild prolongations in prothrombin time and decreases in platelet counts have been observed and neither are important predictors of disease severity or outcomes. 6 Although this triad of increased D-dimer, prolonged thrombin time, and thrombocytopenia could be indicative of disseminated intravascular coagulation (DIC), most patients with COVID-19 do not meet the criteria for DIC according to the International Society on Thrombosis and Hemostasis (ISTH). 7 Despite this low-grade DIC, the rampant inflammatory endothelial cell injury and thrombotic microangiopathies result in activation of the fibrinolytic system and the massive release of endogenous tissue plasminogen activators leading to the high concentrations of D-dimer and mortality rates in patients with severe COVID-19 infection. 5

The incidence of thromboembolic complications among critically ill intensive care unit (ICU) patients with COVID-19 has been reported as high as 69%. 8 9 10 While several studies demonstrate improved outcomes including reduced mortality among patients with COVID-19-induced coagulopathy administered prophylactic anticoagulation therapy with unfractionated heparin (UFH) or low molecular weight heparin (LMWH), others report high rates of thrombotic complications despite the use of pharmacologic prophylaxis. 11 12 As such, therapeutic anticoagulation strategies have been employed and associated with decreased rates of venous thromboembolism (VTE) and improved outcomes. 13 Importantly, the rate of bleeding events appears similar between patients who received therapeutic anticoagulation and those who did not (3 vs. 1.9%, *p* = 0.20). 13

Given the now undeniable link between COVID-19, coagulopathy, and death, it is critical that data to guide optimal anticoagulation strategies be established. In comparison to traditional anticoagulants like UFH, direct oral anticoagulants (DOACs), like apixaban, have the benefit of decreased bleeding complications, lack of required laboratory monitoring, fewer potential drug interactions, less pharmacokinetic (PK) and pharmacodynamic (PD) variability, and superior efficacy. 14 15 16 17 18 Secondary to the more reliable dose–response relationship of anticoagulation with apixaban, use among hospitalized patients has increased in the years since approval. 19 In addition to the general hospitalized setting, apixaban is also being utilized for both initiation and continuation of therapy for critically ill patients admitted to the ICU, 20 21 including those with COVID-19. Apixaban is particularly appealing in the setting of COVID-19 as the twice daily dosing and less frequent monitoring decreases room traffic, medical workers' exposure, and conserves personal protective equipment. Further, apixaban is known to exert anti-inflammatory effects analogous to those of UFH and LMWH 14 by inhibiting plasma-evoked superoxide generation. 15

Despite these numerous purported benefits, to our knowledge, there are no published reports describing the clinical outcomes of apixaban use in critically ill ICU patients with severe COVID-19. 16 17 As such, the purpose of this study was to describe the safety and efficacy outcomes of a cohort of ICU patients with severe COVID-19 respiratory disease treated with therapeutic dose apixaban for COVID-19 at our institution.

## Materials and Methods

### Study Design

**Fig. 1:**
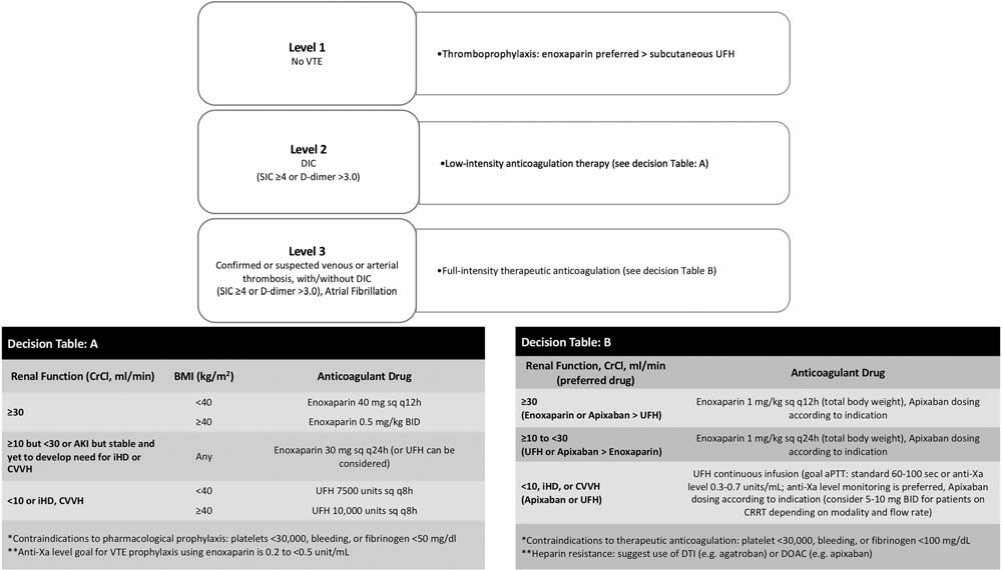
Adapted institutional anticoagulation guideline for the management of COVID-19-induced coagulopathy in ICU patients. AKI, acute kidney injury; aPTT, activated partial thromboplastin time; COVID-19, novel coronavirus disease 2019; DIC, disseminated intravascular coagulation; DOAC, direct oral anticoagulant; SIC, SIC, sepsis-induced coagulopathy; UFH, unfractionated heparin; VTE, venous thromboembolism.

This was a retrospective observational cohort study conducted at the University of Illinois Hospital and Health Sciences System (UI Health), a 495-bed tertiary care academic medical center in Chicago, Illinois, United States. The study was approved by the Office for the Protection of Research Subjects Institutional Review Board with a waiver of consent granted. Adult (≥18 years of age) inpatients admitted to the medical ICU with a confirmed diagnosis of COVID-19 between March and June 2020 who received at least two doses of therapeutic apixaban were eligible for inclusion. The diagnosis of COVID-19 was confirmed via compatible clinical signs and symptoms, radiographic imaging, and a positive nasopharyngeal swab for SARS-CoV-2 ( *M* 2000 REALTI *M* E SYSTEM, Abbott Laboratories, Abbott Park, Illinois, United States) *.* Apixaban was deemed therapeutic if administrated for atrial fibrillation (AFib), treatment of VTE, heparin-induced thrombocytopenia (HIT), line associated-thrombosis, or COVID-19-induced coagulopathy according to our institutional guideline ( Fig. 1 ).

### Data, Outcomes, and Definitions

Data were obtained from the electronic medical record. Baseline was defined as the time of apixaban initiation and data collection continued daily through day 10 of apixaban administration or ICU discharge, whichever was sooner. Variables collected included demographics and baseline characteristics, onset and timing of COVID-19 symptoms and diagnosis, severity of illness, risk of VTE, coagulation parameters, alternative pharmacologic thromboprophylaxis, and organ function. The primary focus of this work was to describe the safety of apixaban for therapeutic anticoagulation in ICU patients with severe COVID-19 disease. Overall safety was defined by the incidence of clinically relevant nonmajor bleeding and major bleeding as defined by the International Society on Thrombosis and Hemostasis (ISTH) in nonsurgical patients. 18 Safety was evaluated from the time of apixaban initiation until the completion of day 10 of therapy or ICU discharge, whichever was sooner. Ten days was chosen to ensure that outcome data were readily available for all patients while allowing for a reasonable assessment of bleeding among ICU patients with COVID-19, given previous data in this population demonstrating a VTE rate of 27% after a median of just 7 days of observation. 9 Secondary outcomes included the incidence of thromboembolic events (VTE or PE), change in coagulation parameters, hospital and ICU length of stay, and mortality.

Additional definitions for disease severity included the DIC score based on the system developed by the ISTH, 19 in which a score ≥5 is considered compatible with overt DIC. The Sepsis-Induced Coagulopathy (SIC) score was based on standard definitions 20 in which a score ≥4 is suggestive of SIC. The sepsis-related organ failure assessment (SOFA) score was defined by the Working Group in Sepsis-Related Problems of the European Society of Intensive Care Medicine. 21 Additionally, the definition of acute respiratory distress syndrome (ARDS) followed the Berlin Definition 22 established by the European Society of Intensive Care Medicine, the American Thoracic Society, and the Society of Critical Care Medicine. Finally, acute kidney injury (AKI) was staged according to the Kidney Diseases: Improving Global Outcomes (KDIGO) criteria. 23

### Statistical Analysis

No formal sample size calculations were performed given the descriptive nature of this investigation. As this was a single-arm study, summary statistics are reported as the number of observations and percentage ( *n* [%]), mean and standard deviation (mean ± standard deviation [SD]) for parametric data, or median and interquartile range (median [IQR]) for nonparametric data.

## Results

During the study period, 21 patients met eligibility criteria. Demographics and baseline characteristics are described in Table 1 . The vast majority of patients (90%) were made up of racial and ethnic minority males (67%) aged 60.9 ± 14.5 years. The rate of relevant concomitant comorbidities was low overall, with the exception of obesity as almost half (42.9%) of the 21 patients had a body mass index (BMI) ≥30 kg/m ^2^ . Table 2 details the disease characteristics of COVID-19 upon patient's admission to the ICU and/or initiation of apixaban. The mean (±SD) time from symptom onset to admission to the hospital or ICU, respectively, was 7.9 ± 4.5 and 6.1 ± 14.6 days while on average patients were admitted to the ICU within approximately 2 days of testing positive for COVID-19. Overall, the majority of patients included had severe COVID-19 respiratory disease as evidenced by the incidence of ARDS (90.5%) and the need for (76.2%) and long median duration of mechanical ventilation (18.5 days). Although, in aggregate, the patients' DIC and SIC scores were not indicative of overt DIC, an SIC score of 3 has been associated with an approximate 19% risk of 30-day mortality while a SOFA score of 8 to 9 is associated with a 26.3 to 33.3% risk of mortality. Further, nearly half (47.6%) of patients qualified for stage-3 AKI and required some form of renal replacement therapy. Finally, mean creatinine clearance also decreased markedly by more than 20 mL/min on average from the time of admission to the ICU to the time of apixaban initiation.

**Table 1:** Demographics and baseline characteristics of 21 ICU patients with severe COVID-19 disease treated with apixaban

Demographic	*n* (%)/mean ± SD/median [interquartile range]
Age (y)	60.9 ± 14.4
Gender (male)	14 (67)
Race	
Black	10 (47.6)
Hispanic/Latino	8 (38.1)
White	2 (9.5)
Asian	1 (4.8)
Weight (kg)	91.1 ± 26
Height (cm)	171.8 ± 12
BMI (kg/m ^2^ )	28.8 [25.4–33.8]
Past medical history	
CHF	7 (33.3)
CVA	3 (14.3)
Chronic lung disease	2 (9.5)
DVT/PE	1 (4.8)
Current malignancy	1 (4.8)
Family history of thrombosis	1 (4.8)
Recent history of estrogen therapy or pregnancy	0 (0)
Bedrest or immobilization ≥72 hours from apixaban initiation	2 (9.5)

Abbreviations: BMI, body mass index; CHF, congestive heart failure; COVID-19, novel coronavirus disease 2019; CVA, cerebrovascular accident; DVT, deep vein thrombosis; ICU, intensive care unit; PE, pulmonary embolism.

**Table 2:** Baseline COVID-19 disease characteristics of 21 ICU patients with severe COVID-19 disease treated with apixaban

Demographic	*n* (%)/mean ± SD/median [interquartile range]
Time from COVID-19 symptom onset to hospital admission (d)	7.9 ± 4.5
Time from COVID-19 symptom onset to ICU admission (d)	6.4 ± 14.6
Time from COVID-19 + test to ICU admission, days	2.1 ± 14.7
Treatment for concurrent non-COVID-19 infection a	20 (95.2)
Mean arterial pressure (mm Hg) b	87.7 ± 13.5
Intravenous vasopressors b	7 (33.3)
Norepinephrine equivalents (µg/kg/min) b	0.02 ± 0.06
ARDS b	19 (90.5)
Mechanical ventilation b	16 (76.2)
Duration of mechanical ventilation (d) b	18.5 [10.3–32.3]
DIC score a	3 [0–2]
SIC score a	3[2-2]
SOFA score a	8.4 ± 3.3
Serum creatinine (mg/dL) b	1.9 ± 1.7
CrCl (mL/min) a	77.4 ± 56.1
CrCl (mL/min) b	57.3 ± 39.4
AKI stage 1	5 (23.8)
AKI stage 2	1 (4.8)
AKI stage 3	10 (47.6)
Renal replacement therapy b	10 (47.6)
iHD	4 (40)
CRRT	6 (60)

Abbreviations: AKI, acute kidney injury; ARDS, acute respiratory distress syndrome; CrCl, creatinine clearance; CRRT, continuous renal replacement therapy; COVID-19, novel coronavirus disease 2019; DIC, disseminated intravascular coagulation; ICU, intensive care unit; iHD, intermittent hemodialysis; SIC, sepsis-induced coagulopathy; SOFA, sepsis-related organ failure assessment.

a Calculated/recorded upon ICU admission.

b Calculated/recorded at the time of apixaban initiation.

**Fig. 2:**
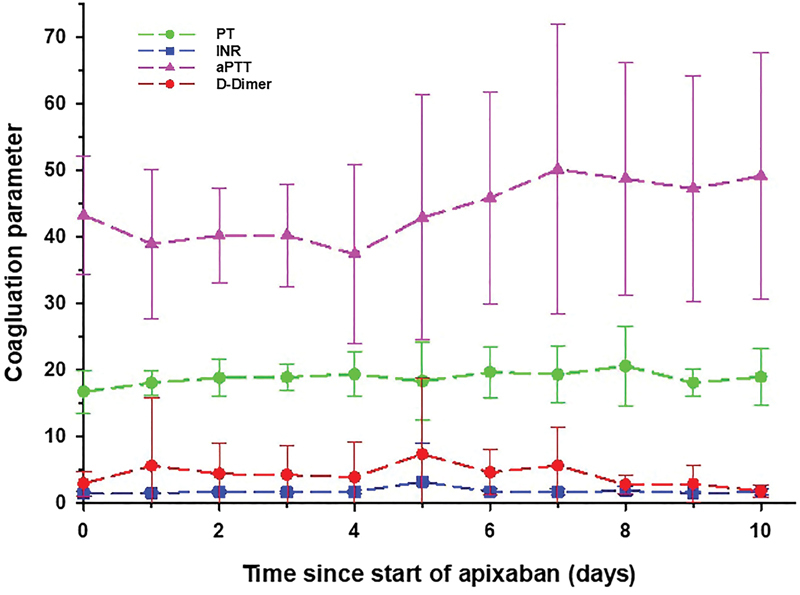
Select coagulation parameters measured over 10 days from the initiation of apixaban in ICU patients with COVID-19. Mean values are displayed with error bars representing standard deviations. aPTT, activated partial thromboplastin time; COVID-19, novel coronavirus disease 2019; ICU, intensive care unit; INR, international normalized ratio; PT, prothrombin time.

Pertinent coagulation parameters along with thromboprophylaxis and therapeutic anticoagulation agents use prior to apixaban are displayed in Table 3 . All but three patients received either prophylactic or therapeutic anticoagulation within 24 hours prior to initiation of apixaban. One-third (33%) received pharmacologic thromboprophylaxis, most commonly with UFH of 5,000 units subcutaneously every 8 hours (57.1%) or enoxaparin of 40 mg subcutaneously every 24 hours (42.9%). The other 11 (52.4%) patients received therapeutic anticoagulation in the 24 hours preceding apixaban initiation; primarily with either UFH (five patients) or enoxaparin (three patients). All 21 patients were transitioned to therapeutic dose apixaban (primarily 5-mg twice a day [BID] in 85.7% of patients) for the treatment of confirmed of suspected VTE (66.7%) or AFib (33.3%). Notably, 11 of the patients who received apixaban for a suspected or confirmed VTE or AFib had been on treatment dose UFH or enoxaparin within 24 hours prior to switching to apixaban. Patients were transitioned to apixaban within a median of 9 days from the time of their COVID-19 diagnosis and therapy was continued for a median of 14 days. At the time of apixaban initiation, all measured coagulation parameters (PT, INR, aPTT, and D-dimer) were elevated above normal with the exception of platelet count ( Table 3 ). These four parameters remained abnormal but stable throughout the 10-day study monitoring period ( Fig. 2 ).

**Table 3:** Coagulation parameters, thromboprophylaxis, and therapeutic anticoagulation of 21 ICU patients with severe COVID-19 disease treated with apixaban

Demographic	*n* (%)/mean ± SD/median [interquartile range]
Pharmacologic thromboprophylaxis within 24 hours prior to apixaban	7 (33.3)
Unfractionated heparin 5,000 units SQ Q8h	4 (57.1)
Enoxaparin 40 mg SQ q24 h	3 (42.9)
Therapeutic anticoagulation within 24 hours prior to apixaban	11 (52.4)
Time from COVID-19 + test to apixaban initiation (d)	9 [0–41]
Indication for apixaban	
Confirmed or suspected VTE	14 (66.7)
Atrial fibrillation	7 (33.3)
Apixaban dose	
5-mg BID	18 (85.7)
10 mg, then 5-mg BID	3 (14.3)
Duration of apixaban therapy (d)	14 [8.5–22.5]
PT	15.6 [15.2–18.6]
INR	1.3 [1.2–1.6]
aPTT	43.8 ± 10.5
D-Dimer	2.4 [1.6–4.1]
Platelet count	217 [138–330]

Abbreviations: aPTT, activated partial thromboplastin time; BID, twice a day; COVID-19, novel coronavirus disease 2019; ICU, intensive care unit; INR, international normalized ratio; PT, prothrombin time; q24hr, every 24 hours; Q8h, every eight hours; SQ, subcutaneous; VTE, venous thromboembolism.

Importantly, despite the severity of illness and the level of renal dysfunction observed among this cohort, none of the patients experienced a thrombosis, major bleed, or bleed requiring discontinuation of apixaban throughout the study period. All but two patients were discharged from the ICU and the hospital, respectively, after a median of 22 [9.5–35] and 25 [20–35] days. Throughout the 10-day study period, four patients succumbed to their COVID-19 disease but were still taking apixaban at the time of their death and did not experience a bleed or thrombosis and therefore their deaths were deemed unrelated to the use of apixaban.

## Discussion

In this cohort of 21 critically ill ICU patients with severe COVID-19 respiratory disease, apixaban was utilized as a modality for therapeutic anticoagulation in patients with a confirmed or suspected VTE or AFib. Notably, 18 (85.7%) of these 21 patients had been receiving anticoagulation with UFH or enoxaparin within at least the 24 hours of preceding the switch to apixaban, including 11 patients who were receiving full-dose therapeutic anticoagulation. 24 Although apixaban was not used solely for COVID-19-induced coagulopathies in this study cohort, the transition to apixaban from UFH or enoxaparin due to confirmed or suspected lack of efficacy is consistent with previous literature demonstrating high rates of thromboembolic complications despite the use of typical pharmacologic thromboprophylaxis agents such as UFH and LMWH. Furthermore, despite the lack of support for DOACs in the most recent CHEST 25 guidelines for the prevention and treatment of COVID-19-induced coagulopathy, we did not observe any adverse events with the use of apixaban including clinically relevant nonmajor or major bleeding events. This is particularly encouraging given the high incidence of renal dysfunction and severe respiratory illness leading to ARDS present in our patient cohort. Finally, over the course of 10 days of apixaban use, there were no suspected or confirmed VTEs, PEs, or strokes due to AFib. This is in contrast to previous data demonstrating a high rate of VTEs (27%) after a median observation time of just 7 days in ICU patients receiving standard thromboprophylaxis. 9 Taken together, these data suggest that apixaban may be a safe and efficacious alternative to UFH or LMWH in hospitalized patients with COVID-19, including those with severe disease. Although larger, randomized trials are needed to confirm these findings, the decreased bleeding complications, lack of required laboratory monitoring, fewer potential drug interactions, less PK and PD variability, and superior efficacy 14 15 16 17 18 compared with traditional agents, such as UFH, will continue to drive the use of therapeutic anticoagulation with apixaban in these high-risk, vulnerable populations in our institution particularly in individuals with an elevated D-dimer >3, clotting of dialysis catheters, unexplained organ failure, or for usual indications of anticoagulation such as established VTE and AFib.

Despite the well-established association between COVID-19, coagulopathy, and mortality, limited data are available regarding optimal prevention and/or treatment modalities and no reliable data exist to guide dosing in this population. A recent analysis of 2,773 COVID-19 patients revealed that the use of therapeutic anticoagulation may be associated with decreased mortality, especially for mechanically ventilated patients. Importantly, the rate of bleeding events was similar between patients who received anticoagulation and those who did not (3 vs. 1.9%, *p* = 0.20). 13 Recent statements by the ISTH and the American Society of Hematology suggest that all hospitalized COVID-19 patients should receive thromboprophylaxis or full-dose anticoagulation if indicated, 7 24 although they make no specific recommendations for which agent to use at what dose. Interestingly, the recent CHEST guideline and expert panel report on the prevention, diagnosis, and treatment of VTE in patients with coronavirus disease 2019 25 recommends against the use of DOACs as pharmacologic thromboprophylaxis in critically ill patients with COVID-19 citing hemodynamic instability, likelihood of drug-drug interactions, and the high incidence of AKI in ICU patients; all of which could contraindicate DOACs. They also state that there is a high risk of rapid clinical deterioration, gastrointestinal dysfunction, and that concomitant therapies, such as antivirals, could significantly affect the PDs of DOACs and thus increase the risk of bleeding. Despite these strong recommendations, no studies comparing different anticoagulants for thromboprophylaxis in acutely ill hospitalized patients with COVID-19 were identified. The only study cited enrolled 12 consecutive patients on DOACs who were hospitalized with severe COVID-19 pneumonia and had DOAC trough levels monitored before and after hospital admission. 26 In this study, the only antivirals administered were lopinavir, ritonavir, or darunavir which are no longer used for SARS-CoV-2 due to their proven lack of efficacy, and all patients also received levofloxacin, azithromycin, and hydroxychloroquine. Only 5 of the 12 patients were on apixaban and the average percentage change in their trough concentrations were 4.5-fold less than that of all the other DOACs (dabigatran, edoxaban, and rivaroxaban) combined. Additionally, no clinical outcomes including major or minor bleeding were reported. Although both efficacy and toxicity have been associated with increased systemic DOAC concentrations, the majority of these data have been observed with DOACs other than apixaban. Only approximately 15% of an apixaban dose is metabolized by CYP3A4 27 and renal clearance accounts for only approximately 27% of elimination. 28 In fact, studies show that even severe renal impairment (CrCl of 15 mL/min) only increased the apixaban AUC by 44% and that dose adjustments of apixaban are not required based on renal function alone. 29 Although apixaban concentrations have been shown to be affected by strong CYP3A4 inhibitors and inducers like ketoconazole and rifampin, respectively, they are largely unaffected by other agents which significantly impact other DOACs. 30 There are no data to suggest concomitant administration of apixaban should be avoided with almost any drug administered for COVID-19 including remdesivir, hydroxychloroquine, levofloxacin, azithromycin, dexamethasone, darunavir, lopinavir, or ritonavir.

## Limitations and Conclusion

Our work adds to the existing data evaluating optimal strategies for anticoagulation in patients with COVID-19-induced coagulopathy and expands on these data by evaluating apixaban in this high-risk ICU population for the first time. This study includes numerous inherent limitations such as the small sample size, retrospective, single-arm nature, and relatively short follow-up period. Despite these limitations, to our knowledge, this is the first set of clinical data evaluating the use of DOACs, specifically apixaban, in ICU patients with severe COVID-19-related disease. Apixaban appeared safe and efficacious in this high-risk patient population and these data encourage future trials attempting to optimize anticoagulation strategies in patients with severe COVID-19.
